# Extraction of the Anisotropic Plasticity of Metal Materials by Using Inverse Analysis and Dual Indentation Tests

**DOI:** 10.3390/ma11010012

**Published:** 2017-12-22

**Authors:** Mingzhi Wang, Jianjun Wu, He Fan, Zengkun Zhang, Hongfei Wu

**Affiliations:** School of Mechanical Engineering, Northwestern Polytechnical University, Xi’an 710072, China; wangmz_nwpu@foxmail.com (M.W.); henwpu@foxmail.com (H.F.); 18292068849@163.com (Z.Z.); WuHongfei321@163.com (H.W.)

**Keywords:** anisotropic plasticity, parameters identification, dimensional analysis, finite element analysis, indentation, inverse analysis

## Abstract

In this paper, a novel inverse computation approach is proposed to extract the anisotropic plasticity parameters of metal materials by using inverse analysis and dual indentation tests. Based on dimensional analysis and extensive finite element (FE) simulations, four independent dimensionless functions are derived to correlate the anisotropic plasticity parameters with material responses in dual indentation tests. Besides, an inverse calculation algorithm is suggested, to estimate the unknown anisotropic parameters of the indented specimens using the information collected from indentation. The proposed numerical approach is applied on a series of engineering materials. Results show that the inverse analysis is ill-posed when only the load-displacement (P-h) curves in dual indentation tests were used. This problem can be effectively alleviated by introducing the pile-up effect as the additional information. The new method is proved to be very effective and reliable.

## 1. Introduction

In nature and synthetic material systems, anisotropic materials are often observed and widely used in industrial products, such as rolled sheets, composites, thin films/coatings and so on [1,2,3]. Since the plastic anisotropy has very obvious influence on the formability and performance of metal materials [4,5], e.g., the in-plane plastic anisotropy is closely related to the tendency of rolled sheets to form ears during drawing [6,7], its mechanical testing is especially important, for the accurate plasticity modeling [8,9]. Traditionally, the plastic anisotropy of metal materials is analyzed by conducting several uniaxial tensile/compression tests along the orthogonal directions. However, this testing method is destructive, and not applicable when the sample volume is exceedingly small [1,3].

In the past few years, with the rapid development of high resolution depth-sensing instrumented equipment, indentation test has been widely used in the extraction of various mechanical properties of materials, e.g., elastic modulus [10], uniaxial stress strain curves of the isotropic materials [11,12,13,14,15], residual stresses [16,17] as well as the material anisotropy [18,19,20,21,22]. One advantage of indentation test is that it is nondestructive [12,13]. Besides, it is well suitable for the extraction of the local mechanical properties from the exceedingly small samples, for which the classical uniaxial tests are not applicable [13,14,15].

Vlassak and Nix fully investigated the elastic anisotropy of single crystals, and revealed that, the “averaged” elastic effects under indentation seems to reduce the sensitivity to measure it [18,19]. To extract the anisotropic parameters of materials using instrumented indentation, researchers resorted to finite element (FE) simulation and inverse analysis, of which some sophisticated optimization algorithms were used. Bocciarelli et al. [20] used conical indentation to calibrate the anisotropic parameters of Hill’s plasticity model, by proper weighting the indentation P-h curve and residual imprint mapping. Nakamura and Gu [21] established a method to estimate the anisotropic elasto-plasticity parameters of the thermally sprayed coatings, of which two indenters with different shapes were considered. It was found that, the P-h curves obtained from these two indenters exhibit opposite behaviors as the modulus ratio changes. Besides, they observed the size and anisotropic effects. Bolzon and Talassi [22] established a novel protocol to extract the elasto-plasticity parameters of anisotropic materials using proper orthogonal decomposition and radial basis functions approximation, of which the numerical computation burden was greatly reduced. However, in these methods [20,21,22], iterative FE simulations are needed in the parameters identification processes, and thus making these protocols not always readily applicable [3,23,24].

Dimensional analysis is a very useful mathematical protocol. It has been widely used to deduce the closed form of universal functions, which are able to effectively capture the indentation responses of materials [3,25]. Besides, it serves as a surrogate model for predicting the indentation shape factors with satisfactory accuracy. Based on dimensional analysis and spherical indentation, Yonezu et al. [3] established a simple framework to evaluate the material plastic anisotropy. In Ref. [3], the concept of representative strain, originally proposed in conical indentation for isotropic materials [11], was extended to spherical indentation on the anisotropic materials. Similarly, Bhat and Venkatesh [25] investigated the computational modeling of the forward and inverse problems in indentation of transversely isotropic power-law hardening materials using dimensional analysis and FE simulation. Although their methods [3,25] are able to extract the anisotropic parameters of materials, the uniqueness of the inverse identified set of anisotropic parameters and its relevant physics are not revealed [12,13,26,27].

In this paper, we proposed a novel inverse computation approach to extract the anisotropic plasticity parameters of metal materials using inverse analysis and dual indentation tests. The advantage of this method is that, the unknown anisotropic parameters of the indented specimens can be readily extracted when the indentation data were inputted into the well-established inverse algorithm. Besides, uniqueness of the inverse identified set of parameters in the relevant questions are carefully analyzed.

## 2. Numerical Approach

### 2.1. Material Model

**Figure 1:**
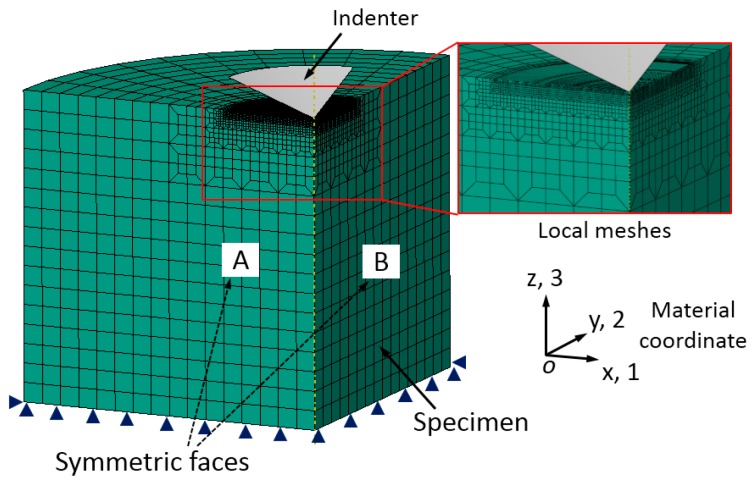
Finite element model, meshes, boundary conditions and material coordinate (right-hand) used in indentation simulation.

To describe the deformation behaviors of anisotropic materials, Hill’s plasticity theory [28] is used, for its relatively simple form and the anisotropic constants are easy to be defined through experiments [28]. The general stress state of this yield criterion is expressed in Equation (1).

(1) $$f\left(\sigma \right)=\sqrt{F{\left(\sigma_{22}-\sigma_{33}\right)}^{2}+G{\left(\sigma_{33}-\sigma_{11}\right)}^{2}+H{\left(\sigma_{11}-\sigma_{22}\right)}^{2}+2L\tau_{23}^{2}+2M\tau_{31}^{2}+2N\tau_{12}^{2}}$$

where, *F*, *G*, *H*, *L*, *M* and *N* are the anisotropic parameters, and they represent the current state of anisotropy [3,28]. These six anisotropic parameters can be determined by using Equation (2). The normal and shear yield stress along three orthogonal axes (e.g., 1, 2 and 3 in the material coordinate defined in Figure 1) are defined as $\sigma_{11}$, $\sigma_{22}$, $\sigma_{33}$ and $\tau_{12}$, $\tau_{31}$, $\tau_{23}$, respectively.

(2) $$F=\frac{1}{2}\left(\frac{1}{R_{22}^{2}}+\frac{1}{R_{33}^{2}}-\frac{1}{R_{11}^{2}}\right);\;G=\frac{1}{2}\left(\frac{1}{R_{33}^{2}}+\frac{1}{R_{11}^{2}}-\frac{1}{R_{22}^{2}}\right);\;H=\frac{1}{2}\left(\frac{1}{R_{11}^{2}}+\frac{1}{R_{22}^{2}}-\frac{1}{R_{33}^{2}}\right);L=\frac{3}{2R_{23}^{2}};\;M=\frac{3}{2R_{13}^{2}};\;N=\frac{3}{2R_{12}^{2}}$$

The six yield stress ratios, $R_{11}$, $R_{22}$, $R_{33}$, $R_{12}$, $R_{13}$ and $R_{23}$ in respectively three normal ($R_{11}$, $R_{22}$ and $R_{33}$) and three shear ($R_{12}$, $R_{13}$ and $R_{23}$) directions are used to quantify the orthogonal anisotropic plasticity, as shown in Figure 1. These six anisotropic constants are inputted by using the POTENTIAL sub-option in ABAQUS software (version 6.14, Dassault, Paris, France) [29]. The *R*-values are defined by using the reference yield stress $\sigma_{Y}$, and the reference shear yield stress $\tau_{Y}$ is defined as $\tau_{Y}=\sigma_{Y}/\sqrt{3}$ according to Von Misses criterion. For the anisotropic materials considered in the present study, the other five *R*-values are maintained as identical at 1, and only $R_{22}$ is varied to simulate the anisotropic plasticity along y (2)-axis as shown by the material coordinate (right-hand) defined in Figure 1. More details about the anisotropic material model studied here can be found in [3,29]. The longitudinal direction (LD) is along y, (2)-axis, and the transverse direction (TD) is along x, (1)-axis. Stress strain curve along transverse direction is defined as the reference input amount, and the longitudinal yield stress is determined by varying $R_{22}$ value using the relation $\sigma_{YL\;}$=$\;R_{22}\sigma_{YT}$.

(3) $$\{\begin{array}{c} \sigma_{L}=E_{s}^{n}\sigma_{YL}^{1-n}\varepsilon_{L}^{n};\;\mathrm{for}\;\sigma_{L}>\sigma_{YL}\\ \sigma_{T}=E_{s}^{n}\sigma_{YT}^{1-n}\varepsilon_{T}^{n};\mathrm{for}\;\sigma_{T}>\sigma_{YT} \end{array}$$

**Figure 2:**
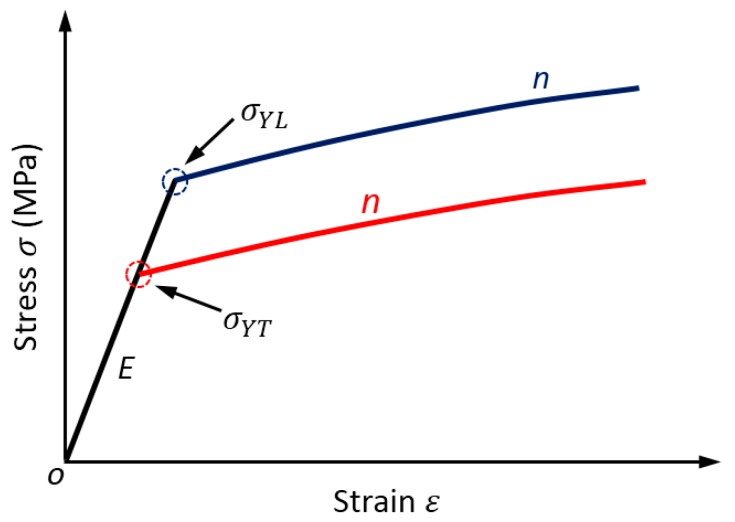
Stress strain curves of anisotropic materials.

The Hollomon hardening model with linear elastic and power law strain hardening plasticity is used to describe the stress strain curves of the anisotropic materials. The strain hardening behaviors of materials is assumed as isotropic using a single strain hardening exponent of “*n*” for both the longitudinal and transverse directions, as expressed in Equation (3). The Hollomon hardening law is able to describe the stress strain behaviors of most engineering materials [30,31]. The elastic modulus is usually isotropic, and it is denoted as $E_{s}$. Figure 2 shows the stress strain curves used to fully characterize the constitutive behaviors of the in-plane anisotropic materials in the present study.

### 2.2. Finite Element Model, Meshes and Boundary Conditions

**Figure 3:**
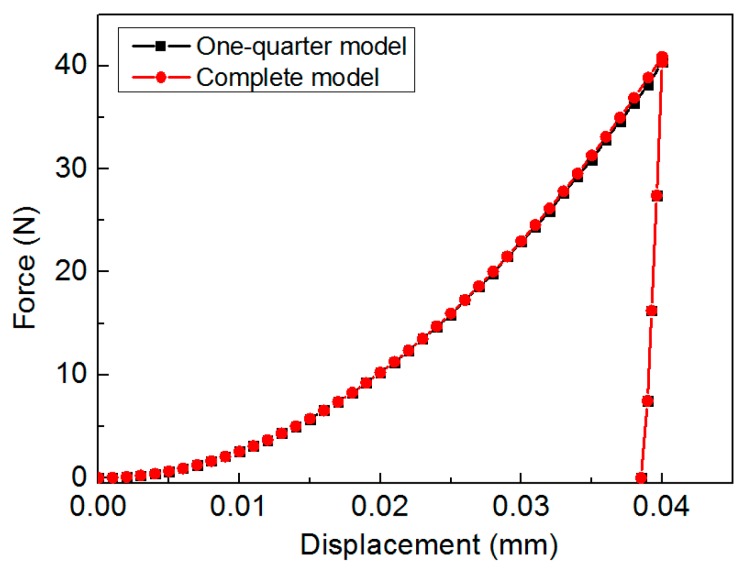
Convergence and effectiveness tests of the one-quarter finite element (FE) model.

The ABAQUS commercial codes [29] were used to simulate the deformation behaviors of anisotropic materials in dual conical indentation tests. The FE model was built in one-quarter to take account of the symmetric structures for both the material and geometry properties in conical indentation, as shown in Figure 1. Two indenters with different inner half angles, 60° and 70°, were used separately. Indenter was assumed as rigid body using R3D4 element type. Specimen was modeled using C3D8R element type. Here, very refined meshes were created in the main deformation region, e.g., the local contact region between indenter and specimen, in order to obtain very accurate numerical results. In this region, the minimum element size was 0.625 μm. The relatively coarse meshes were created in the far away regions, so that the total computation burden can be reduced. The trapezoid meshes were used in the transition area between the two adjacent regions with different element sizes. The design of meshes in the FE model was accomplished with the assistance of Hypermesh software [32], in order to obtain better numerical accuracy and efficiency. Contact friction between surfaces of specimen and indenter was fixed at 0.1, because the contact friction between metals and diamond is around this value [33,34]. Poisson’s ratio of specimen was fixed at 0.3, and it is a minor factor in indentation studies [35]. Height and radius of specimen was 0.64 mm, and this value is large enough to avoid the influence of outer boundary effects. Roller boundary conditions (BC) were applied on the symmetric faces (denoted as A and B in Figure 1) of specimen, and displacement of bottom nodes of specimen was fixed. Indenter was controlled by the displacement up to maximum depth 40 μm, and then the withdrawal of indenter was simulated in one step. The FE model, meshes and boundary conditions described above were the same for the two simulation models with different indenter apex angles. Total number of meshes were 22,940 for specimen, and 1600 and 2000 for the indenters with inner half angles, 60° and 70° respectively. Effectiveness and convergence of the FE model were verified by comparing the computation results with those calculated by a complete model (full 360 degree model). Figure 3 shows the comparison of load-displacement curves calculated from the one-quarter model and the complete model, of which $E_{s}$ = 100 GPa, $\sigma_{YT}$ = 200 MPa, *n* = 0.1, and $R_{22}$ = 1.0. It shows the P-h curves obtained from these two models are nearly the same. Result indicates the effectiveness and convergence of the established one-quarter FE model in the study are good.

### 2.3. Influence of Anisotropic Plasticity on the Material Response in Conical Indentation

**Figure 4:**
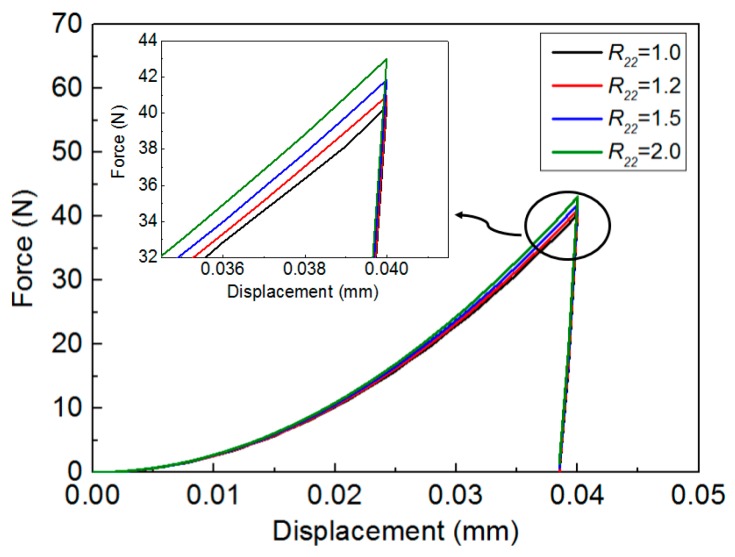
Influence of yield stress ratio $R_{22}$ on the indentation P-h urve ($E_{s}$ = 100 GPa, $\sigma_{YT}$ = 200 MPa, *n* = 0.1, and $R_{22}$ value is varied from 1.0, 1.2, 1.5 to 2.0).

**Figure 5:**
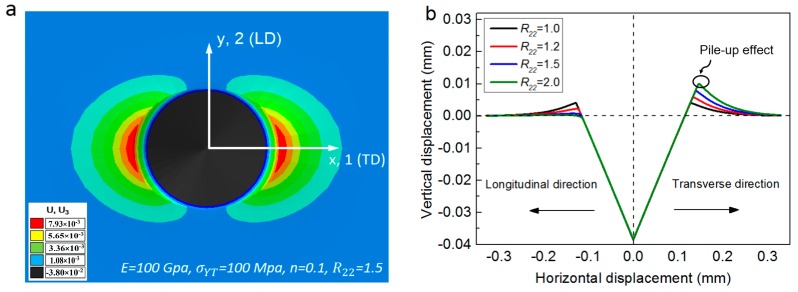
Influence of yield stress ratio $R_{22}$ on the surface deformation of anisotropic materials: (**a**) residual imprint mapping left on the surface of specimen after indenter withdrawal; (**b**) variation of the residual imprints along two orthogonal directions with $R_{22}$ increases.

Figure 4 and Figure 5 show the influence of anisotropic parameter (yield stress ratio $R_{22}$) on the material responses in conical indentation, of which inner half angle of the selected indenter was 70.3°. $E_{s}$, $\sigma_{YT}$ and *n* were fixed, and only $R_{22}$ value was varied from 1.0, 1.2, 1.5 to 2.0. Figure 4 shows the influence of $R_{22}$ on the P-h curve in conical indentation. In this figure, indentation force is monotonously raising with the increase of $R_{22}$ under the same penetration depth. However, the influence of $R_{22}$ on the unloading curve is negligible, because of the pure elastic unloading process.

Figure 5a shows the residual imprint mapping left on the surface of specimen after indenter withdrawal. $E_{s}$, $\sigma_{YT}$, *n* and $R_{22}$ were fixed at 100 GPa, 200 MPa, 0.1 and 1.5, respectively. In Figure 5a, the residual imprint mapping exhibits distinct anisotropy, e.g., the vertical displacement distribution along longitudinal and transverse directions are different. To further reveal this phenomenon, $E_{s}$, $\sigma_{YT}$, *n* are fixed and $R_{22}$ value is varied from 1.0, 1.2, 1.5 to 2.0. The influence of $R_{22}$ value on the residual imprints along longitudinal and transverse directions are plotted in Figure 5b. In this figure, it shows clearly that, the pile-up values along these two orthogonal directions exhibit contrary trends with $R_{22}$ increases. Besides, the direction with lower yield stress is able to exhibit a higher pile-up value.

## 3. The Model

### 3.1. Dimensional Analysis in Indentation of Anisotropic Materials

In this section, dimensional analysis and extensive FE simulations are performed to deduce the forward relationships between anisotropic parameters and material responses in dual conical indentation tests. The loading part of indentation P-h curve can be well approximated by the famous Kick’s law [36,37], as expressed in Equation (4).

(4) $$P=C_{\theta }h_{m}^{2}$$

where, *P* is the indentation force, $h_{m}$ the maximum indentation depth. $C_{\theta }$ is the loading curvature and θ represents the inner half angle of the selected indenters.

In the present study, the material can be fully characterized by five independent parameters: ($E_{s}$, ν, $\sigma_{YT}$, *n*, $R_{22}$), where $E_{s}$ and ν are the elastic modulus and Poisson’s ratio, respectively. In indentation P-h curve, only $C_{\theta }$ is relatively sensitive to the variation of anisotropic parameter $R_{22}$, as it was shown in Figure 4. Therefore, value *C_θ_* is used as the effective indentation shape factor, and it should be the function of indenter geometry and material properties. This function is defined as $f_{\theta }$, as expressed in Equation (5).

(5) $$P_{\theta }=f_{\theta }\left(E_{s},\;\nu ,E_{i},\nu_{i},\sigma_{YT},n,R_{22},h_{m}\right)\;\mathrm{and}\;\theta =60^{{}^{\circ}},70.3^{{}^{\circ}}$$

where, $E_{i}$ and $\nu_{i}$ represent the elastic modulus and Poisson’s ratio of indenter. In all the indentation simulation works, the indenter was assumed as rigid body. So, the reduced modulus $E_{r}$ is used here, and it is defined as $E_{r}=E_{s}/\left(1-\nu_{s}^{2}\right)$ [38]. Using the Π theorem [39], Equation (5) can be converted into the dimensionless form, as expressed in Equations (6) and (7).

(6) $$\frac{P_{60}}{E_{r}h_{m}^{2}}=\frac{C_{60}}{E_{r}}=\Pi_{1}^{60}\left(\frac{\sigma_{YT}}{E_{r}},n,R_{22}\right)$$

and

(7) $$\frac{P_{70.3}}{E_{r}h_{m}^{2}}=\frac{C_{70.3}}{E_{r}}=\Pi_{2}^{70.3}\left(\frac{\sigma_{YT}}{E_{r}},n,R_{22}\right)$$

where, $\Pi_{1}^{60}$ and $\Pi_{2}^{70.3}$ are the dimensionless functions in dual conical indentation tests, with $60^{{}^{\circ}}$ and $70.3^{{}^{\circ}}$ of the inner half angles of the selected indenters.

**Figure 6:**
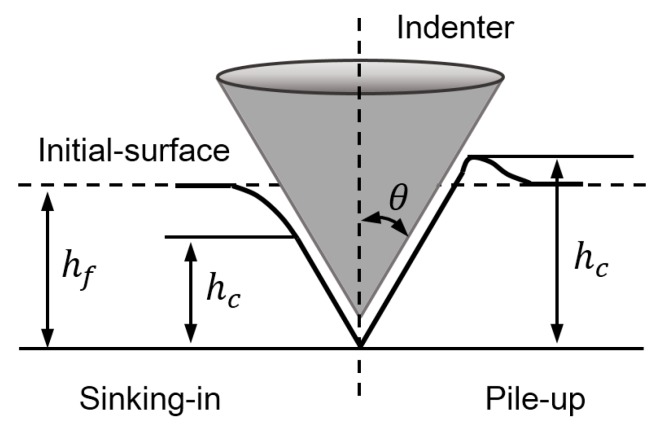
Schematic of the residual imprint in conical indentation.

Surface deformation of materials has long been used as important experiment information in indentation studies [13,40,41,42,43]. Figure 6 shows the schematic of the residual imprint in conical indentation. In this figure, $h_{c}$ is the residual contact depth, and $h_{f}$ the residual indentation depth. When $h_{c}/h_{f}>1$, the material shows pile-up effect. While, the sinking-in effect happens if $h_{c}/h_{f}<1$. In the present study, the material exhibits different pile-up values along the two orthogonal directions, because of plastic anisotropy. Therefore, the surface deformation parameters can be described by the function of indenter geometry and material properties, as expressed in Equation (8).

(8) $$h_{cx}=f_{\theta }\left(E_{r},\sigma_{YT},n,R_{22},h_{m}\right)\;\mathrm{and}\;h_{cy}=f_{\theta }\left(E_{r},\sigma_{YT},n,R_{22},h_{m}\right)$$

where, $h_{cx}$ and $h_{cy}$ represent the residual contact depths along transverse and longitudinal directions, respectively. According to Π theorem [39], Equation (8) can be converted into the dimensionless forms as

(9) $$\frac{h_{cx}^{60}}{h_{m}}=\Pi_{3}^{60}\left(\frac{\sigma_{YT}}{E_{r}},n,R_{22}\right)$$

and

(10) $$\frac{h_{cy}^{60}}{h_{m}}=\Pi_{4}^{60}\left(\frac{\sigma_{YT}}{E_{r}},n,R_{22}\right)$$

In the present study, the four dimensionless functions in Equations (6), (7), (9) and (10) were used to correlate the anisotropic plasticity parameters with material responses in dual conical indentation tests.

### 3.2. Finite Element Analysis and Numerical Regression

The extensive FE simulations were used to deduce the explicit forms of these four dimensionless functions, using the numerical approach developed in Section 2. Total 128 different combinations of material parameters were used in the FE simulations and regression analyses, and they are listed in Table 1. Regression result shows that, the third polynomial basis functions can be used to well approximate all of these four dimensionless functions, and they are expressed as the following

(11) $$\begin{array}{ll} \frac{P_{60}}{E_{r}h_{m}^{2}}=\frac{C_{60}}{E_{r}}=\Pi_{1}^{60} & \left(\frac{\sigma_{YT}}{E_{r}},n,R_{22}\right)=\Pi_{1}^{60}\left(\xi ,\delta ,\eta \right)\\ & =a_{1}+a_{2}\xi +a_{3}\delta +a_{4}\eta +a_{5}\xi \delta +a_{6}\xi \eta +a_{7}\delta \eta +a_{8}\xi^{2}+a_{9}\delta^{2}\\ & +a_{10}\eta^{2}+a_{11}\xi \delta \eta +a_{12}\xi \delta^{2}+a_{13}\xi \eta^{2}+a_{14}\xi^{2}\delta +a_{15}\delta \eta^{2}+a_{16}\xi^{2}\eta \\ & +a_{17}\delta^{2}\eta +a_{18}\xi^{3}+a_{19}\delta^{3}+a_{20}\eta^{3} \end{array}$$

(12) $$\begin{array}{ll} \frac{P_{70.3}}{E_{r}h_{m}^{2}}=\frac{C_{70.3}}{E_{r}}= & \Pi_{2}^{70.3}\left(\frac{\sigma_{YT}}{E_{r}},n,R_{22}\right)=\Pi_{2}^{70.3}\left(\xi ,\delta ,\eta \right)\\ & =b_{1}+b_{2}\xi +b_{3}\delta +b_{4}\eta +b_{5}\xi \delta +b_{6}\xi \eta +b_{7}\delta \eta +b_{8}\xi^{2}+b_{9}\delta^{2}\\ & +b_{10}\eta^{2}+b_{11}\xi \delta \eta +b_{12}\xi \delta^{2}+b_{13}\xi \eta^{2}+b_{14}\xi^{2}\delta +b_{15}\delta \eta^{2}+b_{16}\xi^{2}\eta \\ & +b_{17}\delta^{2}\eta +b_{18}\xi^{3}+b_{19}\delta^{3}+b_{20}\eta^{3} \end{array}$$

(13) $$\begin{array}{ll} \frac{h_{cx}^{60}}{h_{m}}=\Pi_{3}^{60} & (\frac{\sigma_{YT}}{E_{r}},n,R_{22})=\Pi_{3}^{60}\left(\xi ,\delta ,\eta \right)\\ & =c_{1}+c_{2}\xi +c_{3}\delta +c_{4}\eta +c_{5}\xi \delta +c_{6}\xi \eta +c_{7}\delta \eta +c_{8}\xi^{2}+c_{9}\delta^{2}+c_{10}\eta^{2}\\ & +c_{11}\xi \delta \eta +c_{12}\xi \delta^{2}+c_{13}\xi \eta^{2}+c_{14}\xi^{2}\delta +c_{15}\delta \eta^{2}+c_{16}\xi^{2}\eta +c_{17}\delta^{2}\eta \\ & +c_{18}\xi^{3}+c_{19}\delta^{3}+c_{20}\eta^{3} \end{array}$$

(14) $$\begin{array}{ll} \frac{h_{cy}^{60}}{h_{m}}=\Pi_{4}^{60} & (\frac{\sigma_{YT}}{E_{r}},n,R_{22})=\Pi_{4}^{60}\left(\xi ,\delta ,\eta \right)\\ & =d_{1}+d_{2}\xi +d_{3}\delta +d_{4}\eta +d_{5}\xi \delta +d_{6}\xi \eta +d_{7}\delta \eta +d_{8}\xi^{2}+d_{9}\delta^{2}\\ & +d_{10}\eta^{2}+d_{11}\xi \delta \eta +d_{12}\xi \delta^{2}+d_{13}\xi \eta^{2}+d_{14}\xi^{2}\delta +d_{15}\delta \eta^{2}+d_{16}\xi^{2}\eta \\ & +d_{17}\delta^{2}\eta +d_{18}\xi^{3}+d_{19}\delta^{3}+d_{20}\eta^{3} \end{array}$$

where, $\xi =\sigma_{YT}/E_{r}$, δ=n and $\eta =R_{22}$. The fitting parameters in Equations (11)–(14) are listed in Table 2.

**Figure 7:**
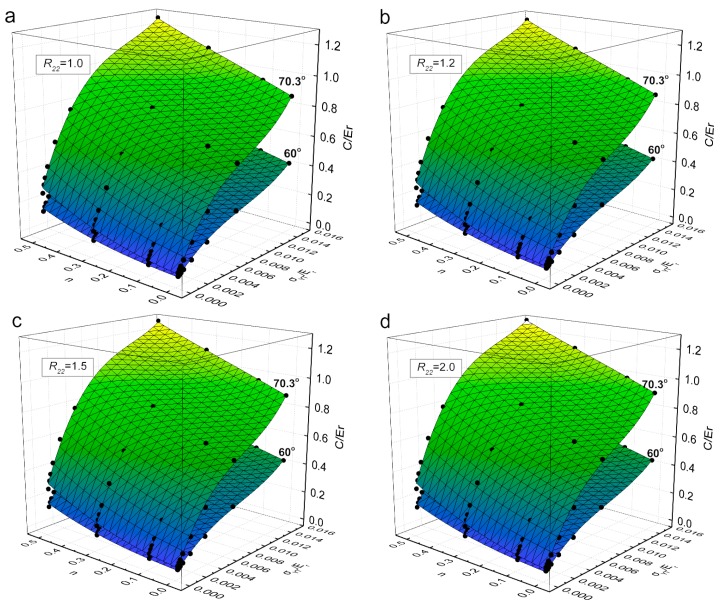
Representative fitting surfaces of dimensionless functions $\Pi_{1}^{60}$ and $\Pi_{2}^{70.3}$ : (**a**) $R_{22}$ = 1.0; (**b**) $R_{22}$ = 1.2; (**c**) $R_{22}$ = 1.5 and (**d**) $R_{22}$ = 2.0.

Figure 7 shows the representative fitting surfaces of $\Pi_{1}^{60}$ and $\Pi_{2}^{70.3}$, as the functions of $\sigma_{YT}/E_{r}$ and *n* for different $R_{22}$ values, and their comparison with the FE data, respectively in Figure 7a for $R_{22}=1.0$, in Figure 7b for $R_{22}=1.2$, in Figure 7c for $R_{22}=1.5$, and in Figure 7d for $R_{22}=2.0$. In Figure 7, the dots (black color) are the data obtained from FE analyses. It shows in Figure 7, that the data obtained from FE analyses can be well represented by the fitting functions of Equations (11) and (12).

**Figure 8:**
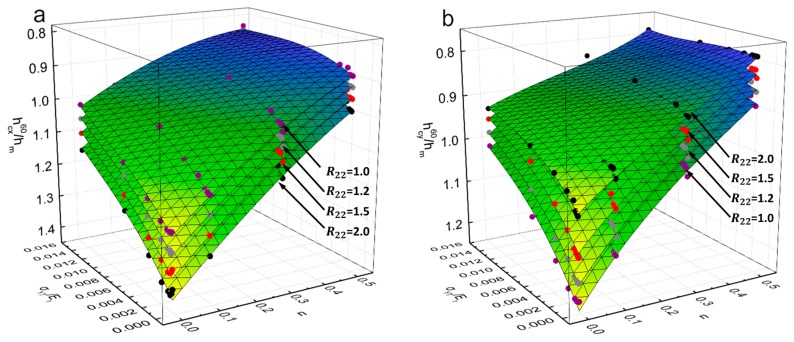
Representative fitting surfaces of dimensionless functions: (**a**)${\;\Pi }_{3}^{60}$; (**b**)${\;\Pi }_{4}^{60}$.

Similarly, Figure 8 shows the representative fitting surfaces of dimensionless functions $\Pi_{3}^{60}$ and $\Pi_{4}^{60}$, and their comparison with the FE data (the dots with different colors), respectively in Figure 8a for $\Pi_{3}^{60}$ and in Figure 8b for $\Pi_{4}^{60}$. For the comparison purpose, the dots with four different colors are used in this Figure, respectively for $R_{22}=1.0$ (Purple), $R_{22}=1.2$ (Gray), $R_{22}=1.5$ (Red) and $R_{22}=2.0$ (Black). As can be seen from Figure 8 that, all the dots are well approximated by the fitting functions of Equations (13) and (14).

### 3.3. Inverse Analysis Algorithm for Predicting the Anisotropic Parameters

**Figure 9:**
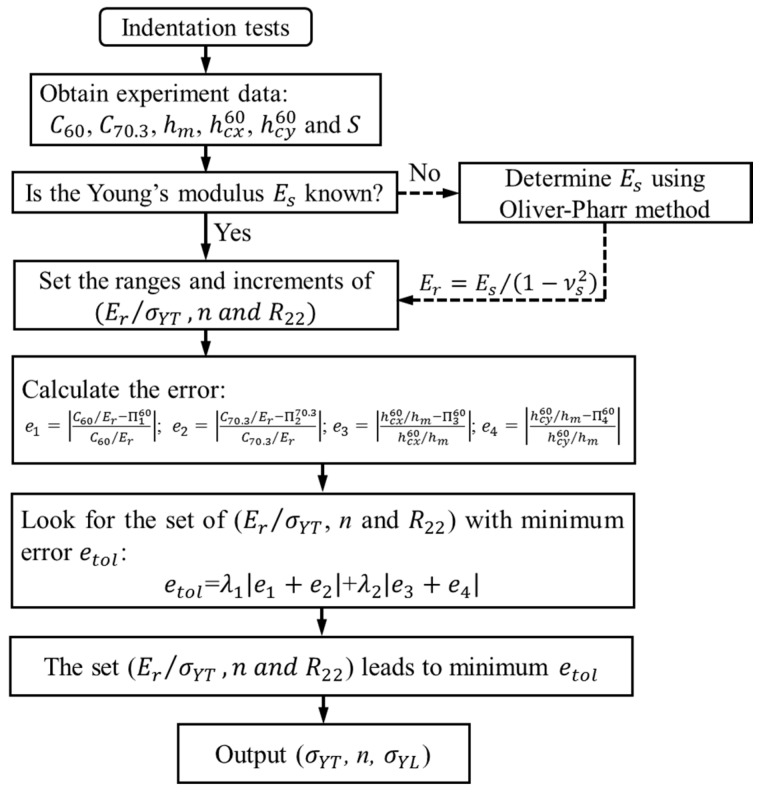
Flow diagram for calculating the unknown anisotropic plasticity parameters using indentation and inverse analysis.

Figure 9 shows the flow diagram for predicting the unknown anisotropic parameters, $\sigma_{YT}$, *n* and $R_{22}$ of the indented specimens using the information collected from indentation. The proposed inverse calculation algorithm is described as the following.

After dual indentation tests, the load-displacement curve and residual imprint left on the surface of specimen are recorded. Therefore, the experimental parameters, $C_{60}$, $C_{70.3}$, $h_{m}$, $h_{cx}^{60}$, $h_{cy}^{60}$ and S are obtained. If the elastic modulus $E_{s}$ is known a prior, the reduced elastic modulus $E_{r}$ can be determined by relation $E_{r}=E_{s}/\left(1-\nu_{s}^{2}\right)$. If $E_{s}$ is unknown, it can be determined by the famous Oliver-Pharr method [10]. The three unknown anisotropic parameters ($E_{r}/\sigma_{YT}$, *n*, $R_{22}$) are varied in a wide range with appropriate increments, and then the errors between “experiment measurements” with respect to those predicted by the four dimensionless functions, $\Pi_{1}^{60}$, $\Pi_{2}^{70.3}$, $\Pi_{3}^{60}$ and $\Pi_{4}^{60}$, are calculated for each combination of the anisotropic plasticity parameters. The summation of the values of these four relative errors from $\Pi_{1}^{60}$, $\Pi_{2}^{70.3}$, $\Pi_{3}^{60}$ and $\Pi_{4}^{60}$ are considered as the total error $e_{tol}$, and the roots of ($E_{r}/\sigma_{YT}$, *n*, $R_{22}$) are determined by finding a best combination, which leads to the minimum value of the total error. In Figure 9, two parameters, $\lambda_{1}$ and $\lambda_{2}$ are the weighting coefficients. When $\lambda_{1}$ = 1 and $\lambda_{2}$ = 0, only the P-h curves in dual indentation tests were used in the inverse analysis. When $\lambda_{1}$ = 1 and $\lambda_{2}$ = 1, the pile-up value will be introduced as the additional information in the inverse analysis. Table 3 listed the anisotropic parameters of four engineering materials.

## 4. Results and Discussion

### 4.1. Uniqueness of the Inverse Identified Set of Parameters Using Indentation and Inverse Analysis

In order to verify the effectiveness of the proposed inverse computation approach, and further interrogate the uniqueness of the inverse problem, using different experiment information (e.g., only the indentation P-h curves or both the indentation P-h curves and pile-up effect) in dual conical indentation tests, we first applied our numerical approach on the Al Castings 242.0-T21. In all the inverse parameters identification processes, the indentation shape factors are obtained by using the “pseudo-experiment”. The indentation response parameters ($C_{60}$, $C_{70.3}$, $h_{cx}^{60}$, $h_{cy}^{60}$ and $h_{m}$) are obtained from FE analysis. Effectiveness of the method is verified by the direct comparison between the FE “Input” anisotropic parameters with those predicted by the proposed inverse computation approach. The advantage of using the “pseudo-experiment” as a replacement of real indentation experiment is that, it is able to circumvent the influence of some uncertain factors, e.g., experiment imprecision and material heterogeneity [12,14,15]. Therefore, we are able to pay close attention to the nature of the inverse problem, e.g., well-posedness or ill-posedness.

The anisotropic parameters of Al Castings 242.0-T21, identified by indentation test and inverse analysis are listed in Table 4 and Table 5, respectively. The increments of material parameters in the inverse algorithm are defined as $\Delta \sigma_{YT}$ = 2.5 MPa, Δn = 0.01 and $\Delta R_{22}$ = 0.001. In the study, two different situations are considered. In situation one, only the P-h curves in dual conical indentation tests are used, and $\lambda_{1}$ = 1 and $\lambda_{2}$ = 0. Result obtained from this situation is listed in Table 4. In situation two, both the indentation P-h curve and pile-up effect are considered, and $\lambda_{1}$ = 1 and $\lambda_{2}$ = 1. Result obtained from situation two is listed in Table 5.

In order to fully investigate the uniqueness of the inverse identified anisotropic parameters, the set of ($\sigma_{YT}$, *n*, $\sigma_{YL}$) with four minimum $e_{tol}$ values (in the ascent order) are recorded, as shown in Table 4 (they are denoted as mat-1, mat-2, mat-3 and mat-4) for situation one and in Table 5 (they are denoted as mat-5, mat-6, mat-7 and mat-8) for situation two, for the comparison purpose. It’s noted that, only the parameter set ($\sigma_{YT}$, *n*, $\sigma_{YL}$) with the minimum $e_{tol}$ value is regarded as the inverse identified material parameters of the indented specimen.

As can be seen from Table 4, that the inverse identified results are scattered in situation one. Four materials with different anisotropic parameters, exhibit very close $e_{tol}$ values, while their anisotropic parameters are completely different. This phenomenon is especially obvious, when the inverse identified parameters are compared with the results reported in Table 5. Results indicate the inverse problem in situation one is ill-posed, although the average values of the inverse identified anisotropic parameters in this situation are close to the FE “Input” amounts. Besides, the Standard Deviation (Std. Dev.) values are relatively large. The Std. Dev. values are 6.61 for $\sigma_{YT}$, 0.014 for *n* and 30.08 for $\sigma_{YL}$. While, in Table 5, it shows that, the four materials have very close $e_{tol}$ values, and they exhibit nearly the same inverse identified anisotropic parameters. So, results proved the inverse problem in situation two is well-posed. In situation two, the average values of the inverse identified anisotropic parameters are very close to the FE “Input” amounts, and the Std. Dev. values are relatively small. The Std. Dev. values are 5.59 for $\sigma_{YT}$, 0.00939 for *n* and 5.07 for $\sigma_{YL}$.

We recall only the P-h curves in dual indentation tests were used in situation one, and the inverse problem in this situation is ill-posed. While, the inverse problem in situation two becomes well-posed when the pile-up effect was introduced as the additional information. In order to reveal the basic physics involved in this phenomenon, the further exploration is made.

**Figure 10:**
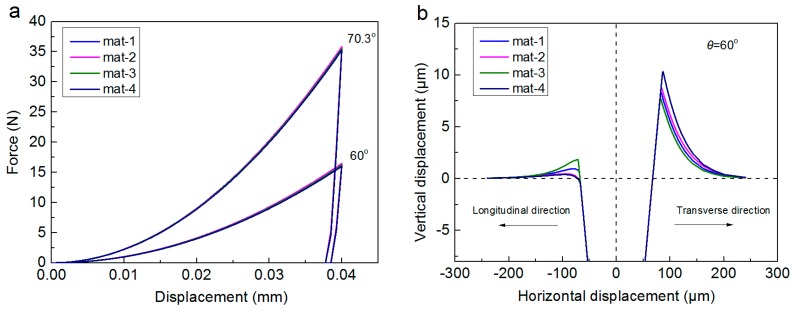
Indentation responses of the four “mystical materials”: (**a**) indentation P-h curves; (**b**) residual imprints along longitudinal and transverse directions.

Figure 10 shows the dual conical indentation responses of four materials (e.g., mat-1, mat-2, mat-3 and mat-4), respectively in Figure 10a for the indentation P-h curves, and in Figure 10b for the residual imprints along longitudinal and transverse directions. It shows clearly in Figure 10a, that the P-h curves of these four anisotropic materials are nearly coincident, and they cannot be uniquely identified by the dual indenters with different apex angles. This explains the reason why the inverse problem in situation one is ill-posed. These four materials can be regarded as the “mystical materials” [27,45], which exhibit different anisotropic parameters, while their P-h curves in dual conical indentation tests are undistinguishable.

However, the pile-up effects of these four “mystical materials” exhibit very obvious differences, as shown in Figure 10b. This explains again why the inverse problem in situation two becomes well-posed when the pile-up effect was considered. Therefore, result in Figure 10 indicates the pile-up effect is very important factor for obtaining the well-posed solution of anisotropic parameters in dual conical indentation tests.

### 4.2. Numerical Verification

**Figure 11:**
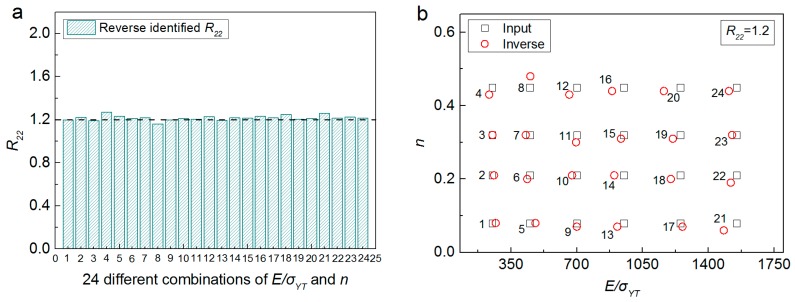
Comparison of 24 different combinations of FE “Input” anisotropic parameters with those identified by using the inverse algorithm: (**a**) $R_{22}$; (**b**) $E/\sigma_{YT}$ and *n*.

**Figure 12:**
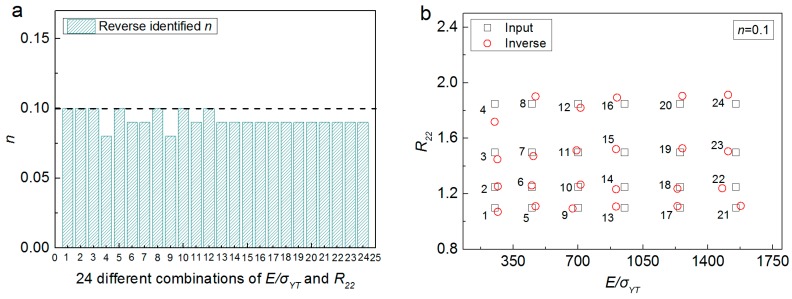
Comparison of 24 different combinations of FE “Input” anisotropic parameters with those identified by using the inverse algorithm: (**a**) *n*; (**b**) $E/\sigma_{YT}$ and $R_{22}$.

The effectiveness of the proposed inverse computation approach is further checked. The indentation response parameters ($C_{60}$, $C_{70.3}$, $h_{cx}^{60}$, $h_{cy}^{60}$ and $h_{m}$) obtained from FE analysis using a wide range of material anisotropic parameters ($E/\sigma_{YT}$, *n* and $R_{22}$), are used as the input data into the inverse calculation algorithm. The inverse extracted anisotropic parameters are compared with those “Inputted” into the FE simulations, and results are shown in Figure 11 and Figure 12, respectively.

Figure 11 shows the comparison between inverse identified anisotropic parameters with those FE “Input” amounts, where $R_{22}$ is fixed at 1.2, $E/\sigma_{YT}$ is varied from 250 to 1550, and *n* is varied from 0.08 to 0.45. Similarly, Figure 12 presented the comparison between inverse identified anisotropic parameters with those FE “Input” amounts, where *n* is fixed at 0.1, $E/\sigma_{YT}$ is varied from 250 to 1550, and $R_{22}$ is varied from 1.10 to 1.85. It can be seen from Figure 11 and Figure 12, that good agreement can be found between the inverse identified anisotropic parameters and those FE “Input” values. The maximum error values are 6.67% for $E/\sigma_{YT}$ (No. 1, Figure 12b), −9.52% for *n* (No. 22, Figure 11b) and 5.9% for $R_{22}$ (No. 4, Figure 11a). Results indicate the proposed numerical approach is effective and reliable.

It is noted that, in the proposed numerical approach, both the P-h curves in dual conical indentation tests were used. The possible reason is that, using the P-h curves of dual indenters is able to give a unique solution of the plastic parameters (e.g., yield stress and strain hardening exponent) of isotropic materials, as reported in the previous literatures [13,27,34,45]. While, in the present study, it was demonstrated that, the inverse problem is still ill-posed when both the P-h curves in dual indentation tests were considered, and this problem was successfully alleviated by introducing the pile-up effect as the additional information. This can be considered as a special situation for the anisotropic materials in the present study, and it is different from the case of isotropic materials reported in the previous literatures [27,34]. Besides, only the pile-up effect in the single indentation (in the study, the pile-up values were obtained using the conical indenter with inner half angle $60^{{}^{\circ}}$) is considered as additional information. Results show the inverse problem becomes well-posed when the pile-up effect is considered. The pile-up effect from a more sharp indentation (inner half angle is $60^{{}^{\circ}}$) is used, because the pile-up effect induced by a sharper indenter is more obvious. Perhaps, the nature of the inverse problem, e.g., well-posedness, may be better if both the pile-up effects in dual indentations were considered [13,45]. However, this needs to formulate six independent dimensionless functions in the inverse algorithm, and it seems not encouraging. Besides, measuring the pile-up values in dual indentation experiments is more complex.

### 4.3. Application on the Engineering Materials

In this section, the proposed inverse computation approach is applied on four engineering materials. The anisotropic parameters of these engineering materials are listed in Table 3. Both the indentation P-h curves and pile-up effect are considered in the inverse algorithm, and $\lambda_{1}$ = 1 and $\lambda_{2}$ = 1. The inverse identified parameters of Al Castings 242.0-T21 has been reported in Section 4.1, so only the inverse identified set of anisotropic parameters of Malleable Iron, Ductile Iron ASTM A 476-70 and Al 6092 17.5 SiC whiskers are listed in Table 6.

**Figure 13:**
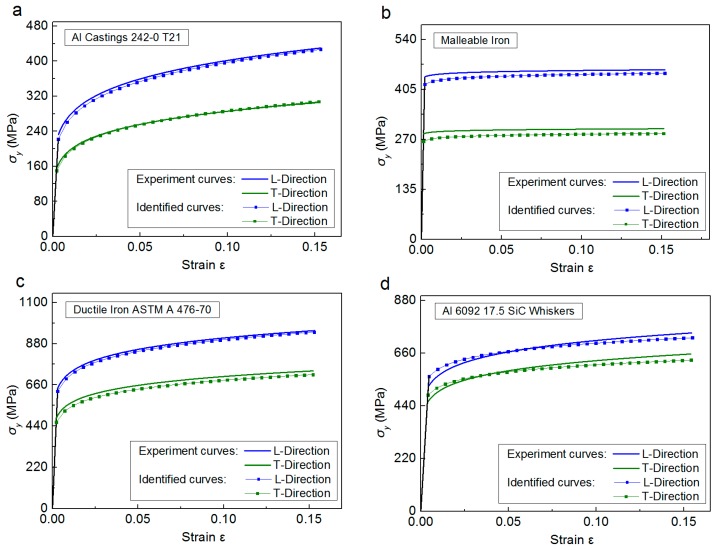
Comparison of the stress strain curves of four anisotropic materials between the FE “Input” values and these obtained from indentation and inverse analysis: (**a**) Al Castings 242.0-T21; (**b**) Malleable Iron; (**c**) Ductile Iron ASTM 476-70; (**d**) Al 6092 17.5 SiC Whiskers.

In Table 6, the anisotropic parameters obtained from dual indentation tests show good agreement with the FE “Input” amounts. The maximum error values are −7.87% for $\sigma_{YT}$ (Ductile Iron ASTM A 476-70), −20% for *n* (Al 6092 17.5 SiC whiskers) and +8.11% for $\sigma_{YL}$ (Al 6092 17.5 SiC whiskers). The stress strain curves obtained from indentation and inverse analysis are compared with those obtained by the representation of Equation (1) using the FE “Input” anisotropic parameters, as shown in Figure 13.

It is noted that, the inverse identified $\sigma_{YT}$ and $\sigma_{YL}$ are very accurate, because their maximum error value is less than 10%. While, the identified *n* shows relatively larger error values, which indicates *n* is more sensitive to some numerical uncertainties, e.g., numerical oscillation and fitting imprecision. In Table 6, the maximum error of *n* is about 20%, e.g., Al 6092 17.5 SiC Whiskers. Here, the FE “input” *n* of Al 6092 17.5 SiC Whiskers is 0.1, while the inverse identified *n* is 0.08. The difference of these two *n* values is 0.01, and it is twice bigger than the value of Δn, 0.01 used in the inverse algorithm. That’s to say, the accuracy of the inverse identified *n* value is also determined by the magnitude of the prescribed increments, $(\Delta \sigma_{YT}$, Δn, $\Delta R_{22})$ in the inverse algorithm. More accurate *n* can be obtained when more refined increments $(\Delta \sigma_{YT}$, Δn, $\Delta R_{22})$ are used, while this will increase the corresponding computation costs greatly, e.g., computation time.

Another important reason for the relatively larger error value of *n* is that, the numerical magnitude of *n* itself is very small, e.g., it is usually less than 0.5. So, its small disturbance will cause obvious relative error, especially when *n* is less than 0.1. For example, the FE “input” *n* of Al 6092 17.5 SiC Whiskers is 0.1, while the inverse identified *n* is 0.08. The difference between 0.08 and 0.1 is 0.02, which is a very small value. While, the corresponding relative error is as large as 20%. Similar situations can also be found in the previous literatures [11,34]. The above two factors explain the reasons why the error of *n* is always relatively larger. So, the established method in the present study still has very good numerical accuracy, and the proposed inverse computation approach is effective and reliable.

In the established dimensionless functions, the Poisson’s ratio is assumed as a constant at 0.3. The Poisson’s ratio of real materials in Table 6 may be larger or smaller than 0.3. While, Poisson’s ratio of common metals is around this value, and it is a minor factor in indentation studies [11,34,35]. Its influence on the indentation responses is very slight, when its value is within 0.25–0.5 [11,27,35]. It is noted that, if the Poisson’s ratio of the indented specimen is extremely small, or even negative, e.g., for some auxetic materials [46,47], the present method should be carefully used.

### 4.4. Discussion

In the present study, we mainly focus on the metal materials which exhibit obvious plastic anisotropy along the longitudinal and transverse directions, while the anisotropy of elastic modulus and strain hardening exponent are slight. In other words, the material studied here exhibits transverse isotropic property [25]. These materials can be found, for the metals which have experienced rolling/extrusion processing, e.g., extruded rod. The anisotropy of these metals arises from the directions/textures of the crystal lattices of grains, and the orientations of the crystal slip systems. More information of the physical origin of anisotropy in metallic materials can be found in Refs [48,49]. Besides, some SiC whisker-reinforced aluminum alloy also exhibit this sort of anisotropy, e.g., SiCw/A6061 [3].

In the study, we used a simplified Hill’s yield criterion, which involves several assumptions. These assumptions help to reduce the complexity of the problem, so that our attention can be focused on the principal characteristics of the studied anisotropic materials, e.g., the difference of yield stress along transverse and longitudinal directions. So, only $R_{22}$ is studied, and the other *R*-ratio values are maintained as identical at 1. It’s noted that, the other *R*-ratio values of the real materials, e.g., the materials in Table 6, may not be completely identical at 1. While their yield stress along transverse and longitudinal directions exhibit major differences. That’s to say, the simplified constructive law in the present study is still a reasonable approximation of the constitutive behaviors of these real materials.

Besides, the real plastic anisotropy of a material can be sometimes far more complex than the simple transverse isotropic one [25]. However, in indentation studies, the uniqueness of the inverse identified set of parameters still remains a scientific challenge, and it determines the practical usefulness of these methods [13,40]. In the study, when only $R_{22}$ is considered in the constitutive model, the non-uniqueness problem also happens if the residual pile-up effect is not introduced. From this point of view, if all these six *R*-ratios are considered, it will complicate the inverse analysis greatly. Besides, the unique solution of the inverse identified parameters is very difficult to be achieved. The elastic anisotropy is another consideration, which is usually physically unavoidable for the metallic crystals, because of lattice orientation. While, in indentation studies, the deformation of materials under indenter is plasticity dominated. That’s to say, the plastic anisotropy domains the elastic anisotropy in conical indentation, and thus the latter is a minor factor. These problems are still open questions, and will be further studied in our future work.

## 5. Conclusions

In this paper, we developed a novel inverse computation approach to extract the anisotropic plasticity parameters of metal materials, based on dimensional analysis and dual conical indentation tests. Dimensional analysis was used to correlate the anisotropic plasticity parameters with the material responses in dual conical indentation tests, and their explicit function forms were established using numerical regression and extensive FE simulations. An inverse calculation algorithm was suggested to predict the unknown anisotropic parameters of the indented specimens using the information collected from instrumented indentation. Effectiveness of the proposed inverse computation approach was verified by its application on a series of engineering materials, and the uniqueness of the inverse identified set of anisotropic parameters was analyzed. Result shows the inverse problem may be ill-posed when only the indentation P-h curves in dual conical indentation tests were used. While, this problem can be effectively alleviated by introducing the pile-up effect as the additional information. Besides, the proposed numerical approach is proved to be very effective and reliable. Lastly, it should be emphasized that the present study mainly focuses on the mathematical development of the inverse algorithm, and theoretical analysis of the nature of the inverse problem, e.g., well-posedness or ill-posedness. Further experimental investigation is very necessary and will be reported in our future work.

## Figures and Tables

**Table 1:** Material parameters used in the 128 FE analyses.

Property	Values
*E* (GPa)	50, 100, 200, 300
$\sigma_{YT}$ (MPa)	100, 200, 400, 800
$E/\sigma_{YT}$	62.5, 125, 250, 500, 1000, 1500, 2000, 3000
*n*	0, 0.1, 0.3, 0.5
$R_{22}$	1.0, 1.2, 1.5, 2.0

**Table 2:** Fitting parameters of the four dimensionless functions, $\Pi_{1}^{60}$ , $\Pi_{2}^{70.3}$, $\Pi_{3}^{60}$ and $\Pi_{4}^{60}$ in Equations (11)–(14).

Number	$a_{i}$($\Pi_{1}^{60}$)	$b_{i}$($\Pi_{2}^{70.3}$)	$c_{i}$($\Pi_{3}^{60}$)	$d_{i}$($\Pi_{4}^{60}$)
1	−4.231 × 10^−3^	1.383 × 10^−1^	8.156 × 10^−1^	1.693
2	5.836 × 10^1^	1.369 × 10^2^	−1.037E × 10^1^	−3.853 × 10^1^
3	−3.104 × 10^−3^	3.667 × 10^−2^	−5.583 × 10^−1^	−1.642
4	−1.288 × 10^−2^	−3.294 × 10^−1^	5.732 × 10^−1^	−6.018 × 10^−1^
5	9.074 × 10^1^	1.380 × 10^2^	1.812 × 10^1^	5.394 × 10^1^
6	2.485	3.709 × 10^−1^	−1.793 × 10^1^	8.383
7	3.909 × 10^−2^	5.938 × 10^−2^	−4.834 ×10^−1^	5.938 × 10^−1^
8	−5.039 × 10^3^	−1.184 × 10^4^	9.044 × 10^2^	1.516 × 10^3^
9	2.937 × 10^−1^	6.555 × 10^−1^	4.262 × 10^−1^	1.552
10	1.022 × 10^−2^	2.287 × 10^−1^	−1.510 × 10^−1^	1.888 × 10^−1^
11	−5.223 × 10^−1^	−4.911	−1.663	−8.119
12	−2.626 × 10^1^	−5.974 × 10^1^	6.249	−1.186 × 10^1^
13	6.471 × 10^−2^	1.767	3.892	5.897 × 10^−1^
14	−4.077 × 10^3^	−5.865 × 10^3^	2.951 × 10^2^	−9.830 × 10^2^
15	−2.092 × 10^−3^	2.761 × 10^−3^	1.200 × 10^−1^	−5.669 × 10^−2^
16	−8.764E × 10^1^	−1.938 × 10^2^	1.851 × 10^2^	−1.827 × 10^2^
17	−4.543 × 10^−3^	−3.425 × 10^−2^	−6.207 × 10^−2^	−4.268 × 10^−1^
18	2.047 × 10^5^	4.573 × 10^5^	−3.351 × 10^4^	−2.923 × 10^4^
19	3.892 × 10^−1^	4.126 × 10^−1^	1.099 × 10^−1^	−8.115 × 10^−1^
20	−2.749 × 10^−3^	−5.150 × 10^−2^	8.181 × 10^−3^	−2.407 × 10^−2^
$R^{2}$	0.999	0.998	0.998	0.997

**Table 3:** Four engineering materials and their anisotropic parameters [25,44].

Materials	$E_{0}$ (GPa)	$\sigma_{YT}$ (MPa)	$\sigma_{YL}/\sigma_{YT}$	*n*
Al Castings 242.0-T21	71.0	155.0	1.50	0.16
Malleable Iron	210.0	285.0	1.54	0.01
Ductile Iron ASTM A 476-70	210.0	483.0	1.33	0.10
Al 6092 17.5 SiC whiskers	121.0	452.5	1.15	0.10

**Table 4:** Material parameters identified from inverse analysis using only the P-h curves in dual indentation tests.

Al Castings 242.0 T21	$\sigma_{YT}$ (MPa)	*n*	$\sigma_{YL}$ (MPa)	$e_{tol}$
Input	155.0	0.160	232.5	-
${P-h}_{60}$ + ${P-h}_{70.3}$				
mat-1	160.00	0.141	243.20	0.0001920
mat-2	150.00	0.158	259.50	0.0002880
mat-3	167.50	0.129	227.80	0.0002900
mat-4	155.00	0.135	302.25	0.0005440
Average	158.13	0.141	258.2	-
Error (%)	2.02	−12.03	11.05	-
Std. Dev.	6.61	0.014	30.08	-

**Table 5:** Material parameters identified from inverse analysis using both the indentation P-h curves and pile-up value.

Al Castings 242.0 T21	$\sigma_{YT}$ (MPa)	*n*	$\sigma_{YL}$ (MPa)	$e_{tol}$
Input	155.0	0.160	232.5	-
${P-h}_{dual}$ + ${\mathrm{Pile}-\mathrm{up}}_{60}$				
mat-5	142.50	0.181	221.16	0.013824
mat-6	142.50	0.181	221.16	0.013825
mat-7	142.50	0.181	221.30	0.013838
mat-8	142.50	0.181	221.02	0.013855
Average	142.50	0.181	221.16	-
Error (%)	−8.06	13.13	−4.88	-
Std. Dev.	5.59	0.00939	5.07	-

**Table 6:** Comparison of the anisotropic parameters between FE “Input” amounts with those obtained from inverse analysis and dual indentation tests.

Materials	$\sigma_{YT}$ (MPa)	*n*	$\sigma_{YL}$ (MPa)
Malleable Iron			
Input	285.0	0.010	438.9
Indentation	265.0	0.009	418.7
Error (%)	−7.02	−10.00	−4.60
Ductile Iron ASTM A 476-70			
Input	483.0	0.100	642.4
Indentation	445.0	0.110	605.2
Error (%)	−7.87	+10.00	−5.79
Al 6092 17.5 SiC Whiskers			
Input	452.5	0.100	520.4
Indentation	485.0	0.080	562.6
Error (%)	+7.18	−20.00	+8.11

## Nomenclature

*F*, *G*, *H*, *L*, *M* and *N*	Anisotropic constants
$R_{11}$, $R_{22}$,$\;R_{33}$,$\;R_{12}$,$\;R_{13}$ and $R_{23}$	Six anisotropic yield stress ratios
$E_{s}$	Specimen’s elastic modulus
$\sigma_{YL}$	Longitudinal yield stress
$\sigma_{YT}$	Transverse yield stress
*n*	Strain hardening exponent
$E_{i}$	Indenter’s elastic modulus
$\nu_{i}$	Indenter’s Poisson’s ratio
$E_{r}$	Reduced elastic modulus
$h_{m}$	Maximum indentation depth
$h_{f}$	Maximum residual depth
P	Indentation load
$h_{cx}$	Residual contact depth along transverse direction
$h_{cy}$	Residual contact depth along longitudinal direction
θ	Inner half angle of indenter
$C_{\theta }$	Loading curvature
